# Peculiar properties of tuber starch in a potato mutant lacking the α-glucan water dikinase 1 gene *GWD1* created by targeted mutagenesis using the CRISPR/dMac3-Cas9 system

**DOI:** 10.5511/plantbiotechnology.23.0823a

**Published:** 2023-09-25

**Authors:** Mariko Ohnuma, Kosuke Ito, Karin Hamada, Ami Takeuchi, Kenji Asano, Takahiro Noda, Akira Watanabe, Akiko Hokura, Hiroshi Teramura, Fuminori Takahashi, Hiromi Mutsuro-Aoki, Koji Tamura, Hiroaki Shimada

**Affiliations:** 1 Department of Biological Science and Technology, Tokyo University of Science, Katsushika, Tokyo 125-8585, Japan; 2 Division of Large-Scale Upland Farming Research, Field Crop Breeding Group, Hokkaido Agricultural Research Center, National Agriculture and Food Research Organization (NARO), Kasai, Hokkaido 082-0081, Japan; 3 Department of Applied Chemistry, Tokyo Denki University, Adachi, Tokyo 120-8551, Japan

**Keywords:** freeze and thawing, potato (*Solanum tuberosum* L.), starch phosphorylation, syneresis, targeted mutagenesis

## Abstract

Glucose chains in starch are phosphorylated and contribute to structural stabilization. Phosphate groups contained in starch also play a role in retaining moisture. α-Glucan water dikinase 1 (GWD1) is involved in the phosphorylation of glucose chains in starch. In this study, we generated potato mutants of the *GWD1* gene using the CRISPR/dMac3-Cas9 system. Observation of the phenotypes of the *GWD1*-deficient mutants revealed their physiological roles in tuber starch formation. The 4-allele mutants showed growth retardation and a delay in tuber formation. A significant decrease in phosphorus content was detected in the tuber starch of the *gwd1* mutant. This mutant starch showed a higher amylose content than the wild-type starch, whereas its gelatinization temperature was slightly lower than that of the WT starch. The peak viscosity of the mutant starch was lower than that of the WT starch. These observations revealed that the starch of the *gwd1* mutants had peculiar and unique properties compared to those of WT, *sbe3* and *gbss1* mutant starches. The amount of tissue-released water due to freeze–thawing treatment was determined on tubers of the *gwd1* mutant and compared with those of WT and the other mutants. Significantly less water loss was found in the *gwd1*, *sbe3* and *gbss1* mutant tubers than in the WT tubers. Our results indicate that the *GWD1* gene is not only important for potato growth, but also largely effective for the traits of tuber starch.

## Introduction

Potato is one of four major crops whose annual production is more than 300 million tons worldwide. Potato is widely consumed not only as an edible food but also in large quantities for industrial applications (Hofvander et al. 2022). Potato develops tubers where a large amount of starch is stored. Potato tuber starch is known to have high viscosity compared to starches produced from other plants. This property is suitable in industrial applications such as additives in paper products and adhesives (Huang et al. 2014).

The starch of potato tubers is composed of 20–30% amylose and 70–80% amylopectin (Kuipers et al. 1994). Potato starch has been reported to contain approximately 7.8 nmol of phosphate/mg of starch (Parra and Stevens 2000). Starch granules contain many ester-bound phosphates due to phosphorylation of glucose chains in starch. The amount of phosphate largely affects the viscosity of gelatinized starch and contributes to stabilization of the starch structure because of its close link to the chains (Nielsen et al. 1994).

Potato tubers with a high phosphate content in starch tend to generate many drips by freeze–thawing. This phenomenon is called syneresis, and the water retained in the starch separates and exudes (Nishide 1966). Thus, starch with low syneresis fares better upon freezing (Wang et al. 2022). Potato starch is often used as an additive in pastes such as fish cakes and fish pastes, and the application of starch with low moisture content is expected to contribute to the improvement of water retention and food taste.

The addition of phosphate occurs on the 6-hydroxyl group of glucose in the starch chains. This modification has been reported to be catalyzed by α-glucan water dikinase 1 (GWD1) (Mahlow et al. 2016). Inactivation of GWD1 results in a decrease in the amount of phosphate groups in starch. Potato starch with low phosphate content is thought to show less syneresis during the freeze–thawing process and is expected to be suitable for cryopreservation (Ramadan and Sitohy 2020).

A representative potato cultivar has an autotetraploid genome. To obtain a potato mutant, it is necessary to introduce the desired mutation in all four alleles. Genome editing of potato requires a very powerful tool that efficiently achieves the targeted mutations in all four alleles. A genome-editing tool, CRIPSR/dMac3-Cas9, utilizing the rice translation enhancer dMac3 and three guide RNAs, can introduce any mutant with high efficiency. Using this system, we have obtained potato mutants of *GBSS1* and *SBE3* genes that were involved in starch biosynthesis (Kusano et al. 2018; Takeuchi et al. 2021). In this study, we report the creation of a potato mutant in which the *GWD1* gene was disrupted. Furthermore, we show the phenotypes of the obtained mutants, compare with those of other starch mutants, and discuss the functions of this gene in the formation of tuber starch.

## Materials and methods

### Plant material, tissue culture and plant growth conditions

The *Solanum tuberosum* L. cv. Sayaka was used. Potato plants were cultured on medium containing an MS basal salt mixture (Fuji Film Wako, Tokyo, Japan) (Murashige and Skoog 1962), 3% sucrose, and 0.3% Gelrite (Fuji Film Wako) with the pH adjusted to 6.0. Plants were grown under long-day conditions with 16 h light and 8 h dark at 23°C in a growth chamber. The regeneration of potato plants was performed as described previously (Ohnuma et al. 2020). Regenerated potato plants were cultivated using a growth room. They were grown under 16 h light (200–400 µmol m^−2^ s^−1^) and 8 h dark conditions at 23°C in a growth chamber. Tubers were harvested from wild-type and transformed potatoes, which were grown at 22°C under 16 h light and 8 h dark conditions in a growth chamber.

### Plasmid construction

**Figure 1:**
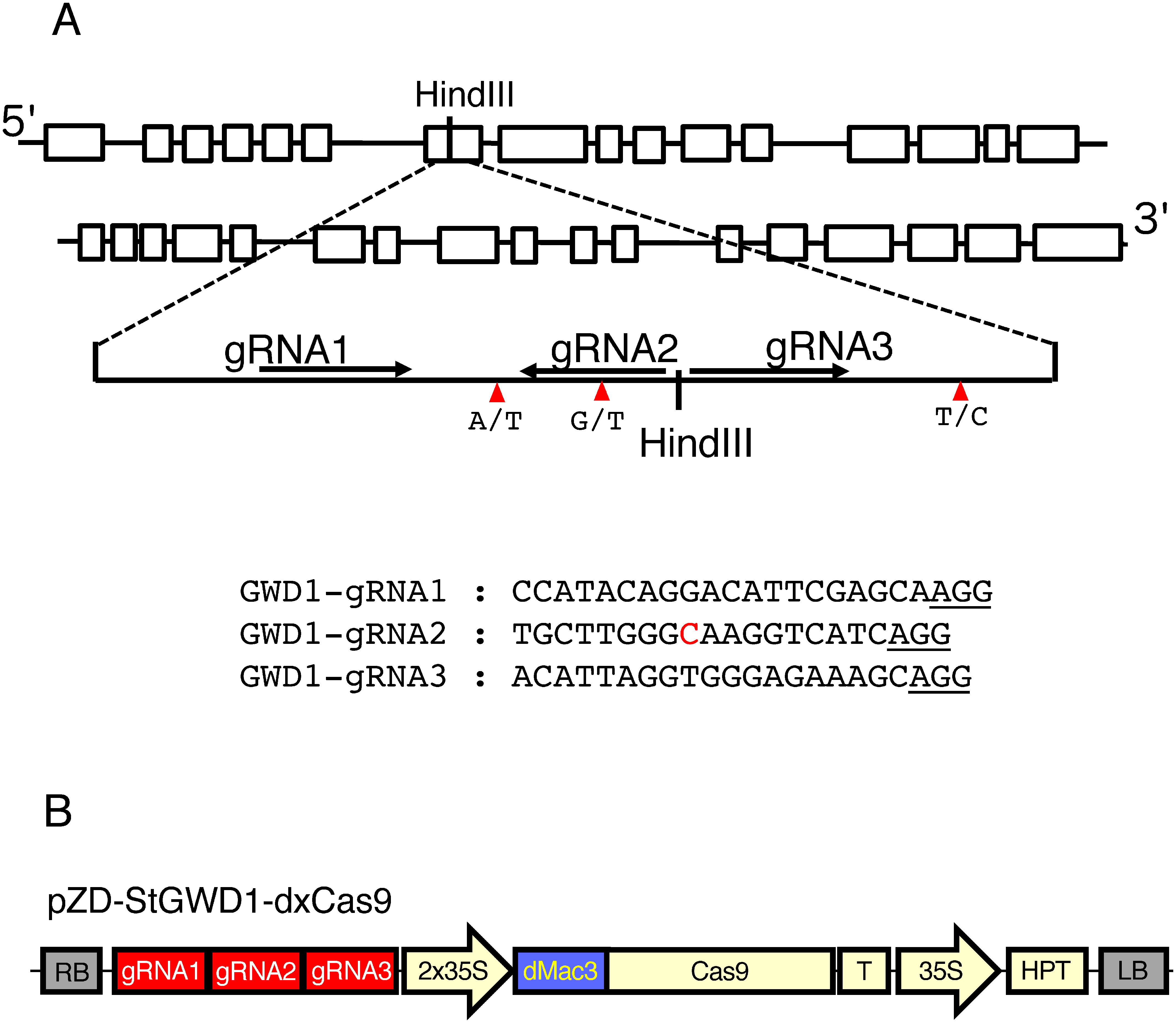
Figure 1. Structure of the potato *GWD1* gene and location of the gRNAs used for targeted mutagenesis. (A) Schematic representation of the *GWD1* gene. Exons are indicated by boxes. The HindIII site used for detection of targeted mutations by CAPS analysis is shown. The region containing the target sequences is indicated below. Arrows indicate the positions of the gRNAs. Single nucleotide differences in the target region are indicated by triangles. The lower panel describes the nucleotide sequences of the gRNAs. Red colored C indicates the polymorphic sequence in the genome WT-A and WT-B of the genome, which is replaced with A in WT-C and WT-D. PAM sequences are underlined. The detailed nucleotide sequence of this region is shown in Supplementary Figure S1. (B) Structure of pZD-StGWD1-dxCas9. gRNA1, gRNA2 and gRNA3: gRNA genes including AT-U6 promoter and gRNA scaffold, 2x35S and 35S: CaMV 35S promoter region, dMac3: dMac3 enhancer sequence, Cas9: coding region of the Cas9 nuclease, T: terminators of pea *3A* and rice *actin 1* genes, HPT: hygromycin resistance gene, and RB and LB: right and left borders.

The plasmid for CRISPR/Cas9 was constructed according to Kusano et al. (2018). The DNA fragments corresponding to gRNAs in the target sequences (Figure 1A) were chemically synthesized and inserted into the BbsI sites of the appropriate guide RNA vectors. The resultant gRNA genes were located between the AtU6-26 promoter and the gRNA scaffold sequence. They were introduced into pBS_GwIsceI using the multisite-Gateway system (Invitrogen, Carlsbad, CA, USA). The pBS_GwIsceI plasmid had the regions for the Gateway attR1 and attR2 sites lying between the two I-SceI sites (Kusano et al. 2018). The resultant plasmid contained the fragments corresponding to the tandem gRNA genes between two I-SceI sites. The gene fragments were digested with I-SceI and then introduced into the I-SceI sites of the pZD-dxCas9 plasmid (Kusano et al. 2018) to generate CRISPR/Cas9 vectors containing three gRNA genes.

### Plant transformation

Potato was transformed using the Agrobacterium-mediated procedure using the *A. tumefaciens* EHA105 strain as described previously (Takeuchi et al. 2021). Callus induction and plant regeneration were performed on a plate of 3C5ZR medium (Sheerman and Bevan 1988) containing 0.3% Gelrite (Fuji Film Wako) supplemented with 5 mg l^−1^ hygromycin B (Fuji Film Wako) and cultured for 2 months. Regenerated plants were assessed by detecting the transgene with PCR using the Cas9-S (5′-GGCGTAAGAATAGAATCTGTTAT-3′) and Cas9-T (5′-GACAGCGCTATCAGATTTCCAA-3′) primers, which amplified part of the *Cas9* gene sequence. Genome-edited potato plants were cultivated in a plant cultivation room.

### Cleaved amplified polymorphic sequence (CAPS) analysis

Genomic DNA was prepared from potato leaves using the RED Extract N-Amp™ Plant PCR kit (Sigma-Aldrich, St. Louis, USA). The 875-nt region in the potato *GWD1* gene, in which the sequences of the target sites of the guide RNAs were contained, was PCR-amplified using the StGWD1_CAPS_Fw (5′-CTATCCTCCACTGCTGTTAGAC-3′) and StGWD1_CAPS_Rv (5′-TCTTCCCAGAGGACTTTGCTACC-3′) primers that were derived from the regions in exon 7. The amplified fragment was digested with HindIII. The wild-type gene has a HindIII site in the corresponding region. Therefore, we identified mutant alleles as those where the amplified fragment was no longer digested by HindIII. The number of mutant alleles among the four genes was estimated by the ratio of undigested fragments to digested fragments.

### DNA sequence analysis

The 0.5-kb region of the potato *GWD1* gene that contained the target sites of the guide RNAs was PCR-amplified using the StGWD1_seq_Fw (5′- AAGGAAAAAAGCGGCCGCCTATCCTCCACTGCTGTTAGAC-3′) and StGWD1_seq_Rv (5′-CCCGATATCGCAACTCATCAAGGGTAATGCC-3′) primers. Then, the amplified fragment was inserted into the pTA2 vector (TOYOBO, Osaka, Japan). The resultant plasmids were used for nucleotide sequence analysis.

### Preparation of potato starch

Potato starch was prepared as described previously (Noda et al. 2004b). The diced potato tuber (approximately 100 g) was homogenized in a mixer with 400 ml water. The slurry was successively filtered through 150 and 106 µm metallic sieves, allowing most of the starch granules to pass. The filtration (starch suspension) was allowed to stand for 2 h. The starch granules were recovered from the extraction by decantation. The remaining starch pellets were washed with water three times, and then dried at room temperature. The purified starch samples were stored at 4°C.

### Determination of the properties of tuber starch

Amylose content, thermal properties (by differential scanning calorimetry; DSC), pasting properties (by rapid visco analyzer; RVA), and resistant starch content were determined by previously described methods (Noda et al. 2019, 2004b). The thermal properties of 30% starch suspension (dry-weight basis, w/w) were determined using a DSC 6100 (Seiko Instruments, Tokyo, Japan), in accordance with the procedures of Noda et al. (2004b). The paste viscosity of 4% (w/w) starch suspension (dry-weight basis, w/w) was determined using an RVA-4 (Newport Scientific Pvt., Ltd., Warriewood, NSW, Australia) as previously reported (Noda et al. 2004b). The resistant starch (RS) content, which corresponds to the ratio of RS to total starch, of native starches was analyzed using a Megazyme Resistant Starch Assay Kit, 05/2008 (Megazyme International Ireland Ltd., Co., County Wicklow, Ireland) in accordance with AOAC method 2002.02 (McCleary and Monaghan 2002). The determinations were performed in triplicate. The data were statistically analyzed using Dunnett’s multiple comparison test.

### Amounts of phosphorus in tuber starch

The phosphorus concentration in starch was determined by fluorescent X-ray analysis using the standard curve method (Otaka et al. 2014; Watanabe et al. 2021). The standard curves for phosphorus were constructed using the representative plant certified reference materials, apple leaves (NIAS SRM 1515), pepperbush (NIES CRM No.1), Chlorella (NIES CRM No.3), and tea leaves (NIES CRM No.23). Tuber starch was lyophilized, and the dried starch sample was ground using a mixer mill (MM400, Retsch, Haan, Germany). Three hundred milligrams of the starch powder was used to make a 20 mm diameter tablet using a manual hydraulic press (GS15011; Specac Ltd., Orpington, UK) at 9 tons of pressure for 10 min. The obtained tablets were provided for the analysis of phosphorus molecules and other elements by an energy dispersive X-ray fluorescence (XRF) spectrometer (Epsilon 5; Malvern Panalytical, Spectris, The Netherlands). The phosphorus concentrations were compared between the wild type and mutants and are indicated by the values relative to that of the WT. The data were statistically analyzed using Dunnett’s multiple comparison test.

### Determination of the amount of water lost from tubers due to freeze–thawing

Wild-type and mutant potato tubers were cut into pieces of approximately 10 g each. Each section was weighed and sufficiently heated in a microwave oven. After cooling naturally, they were wrapped and stored at −20°C for one month. After this treatment, the frozen potato sections were naturally thawed and wrapped in a polymer absorbent sheet (disposable diaper sheets for pets). They were placed under a weight of 4 kg and held for 7 days to allow the polymer absorbent sheet to absorb the moisture released from these tuber sections. With these treatments, the amount of tissue-released water of potato tubers due to freeze–thawing was determined. The weight of the tuber sections was measured at each stage of these treatments. The relative values of the final weight to those of the initial weight of the tuber sections was determined as the ratio of released water from the potato tubers, and they were compared between WT and mutant potato tubers. As a control, sections of wild-type and mutant potato tubers were prepared, heated, and wrapped in polymer absorbent sheets without freeze–thawing, and the amount of tissue-released water from the tubers was measured. These values were compared between the wild type and mutants. The data were statistically analyzed using Dunnett’s multiple comparison test.

## Results

### Determination of the nucleotide sequences of the potato *GWD1* gene and the gRNAs of the target region

The nucleotide sequence of the potato *GWD1* gene has been deposited in the GenBank genome database (Acc. no. JQ388473) and Spud DB (PGSC0003DMT400019845). This gene consists of 33 exons (Uitdewilligen et al. 2022). For the target mutagenesis of the *S. tuberosum GWD1* gene, we selected three regions, gRNA-1, gRNA-2 and gRNA-3, corresponding to the sequences within the seventh exon of the *GWD1* gene (Figure 1A). We analyzed the nucleotide sequence around these gRNA target sites of the *GWD1* gene in the Sayaka genome because no precise nucleotide sequence of Sayaka GWD1 has been determined. We found several differences in the nucleotide sequence to classify four alleles, named WT-A, WT-B, WT-C and WT-D, due to nucleotide polymorphisms (Figure 1A and Supplementary Figure S1). In gRNA-2, there was a G-to-T nucleotide substitution in WT-C and WT-D from those of WT-A and WT-B. Other gRNAs were identical in nucleotide sequence among them. These gRNAs were used for the generation of CRISPR/Cas9. In the region of the target sequences, there was a HindIII site, which facilitated the detection of targeted mutations by CAPS analysis (Figure 1A).

### Generation of potato *gwd1* mutants

Using CRISPR/dMac3-Cas9, we constructed pZD-StGWD1-dxCas9, which contained three gRNA genes (Figure 1B). Using this method, the CRISPR/Cas9 gene was introduced into the stems of potato plants. Among the regenerated potato transformants, mutants of the *GWD1* gene were investigated. There is a HindIII site in or near the target sites, and therefore, the occurrence of targeted mutations can be detected by disruption of this HindIII site. The DNA fragment containing the target site was PCR-amplified, and CAPS analysis was carried out by HindIII cleavage.

**Figure 2:**
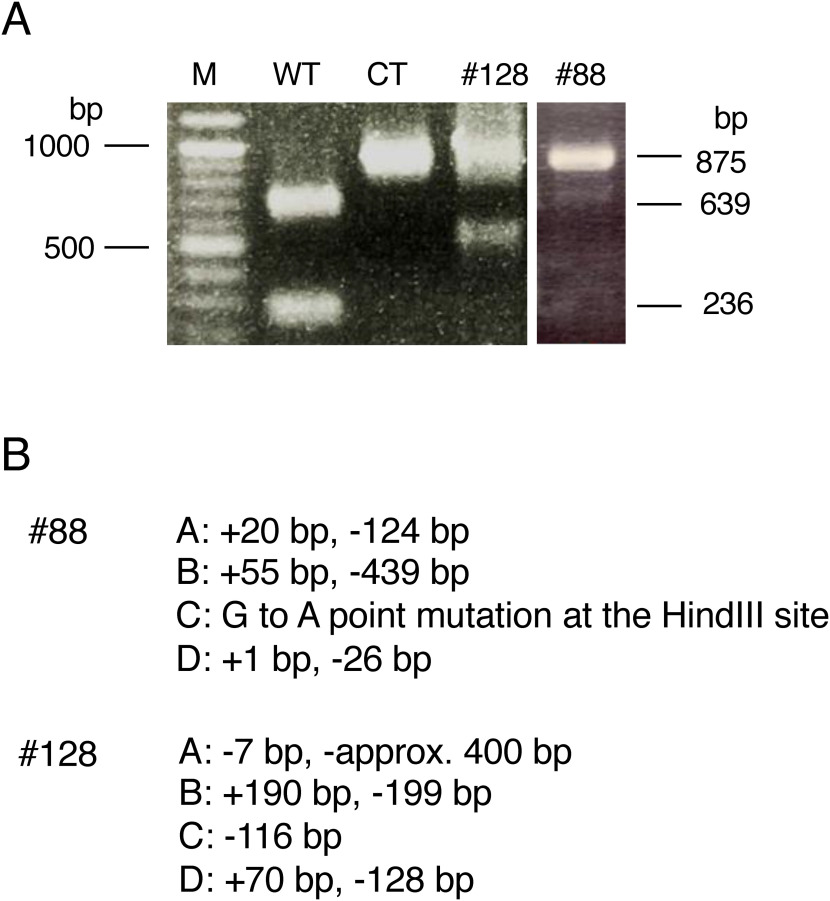
Figure 2. (A) CAPS analysis of the regenerated potato lines #88 and #128. HindIII-digested PCR-amplified fragments are shown. M: size markers, WT: PCR-amplified and HindIII-digested WT fragment. CT: PCR-amplified fragment of the region around the target site in the wild-type gene. The sizes shown on the right side correspond to the PCR-amplified fragment and its HindIII-digested ones. (B) Mutant alleles of the lines #88 and #128. Numbers of nucleotide insertion and deletion that occurred in each of WT-A to WT-D are indicated. Nucleotide sequences are shown in Supplementary Figure S1.

Potato, *S. tuberosum*, has a tetraploid genome in which genome-editing events may occur. Introducing CRISPR/Cas9 resulted in 93 potato transformants (Supplementary Figure S2). The frequency of generated mutations was estimated by the ratio of the HindIII-uncleaved fragments to the amplified fragments. CAPS analysis indicated that 67 transformants (72%) contained mutant genes (Table 1). Among them, two transformants (#88 and #128) were suggested to be mutants that had mutations in all four alleles of the *GWD1* gene (Figure 2A). Ten transformants (11%), 20 transformants (21%) and 35 transformants (38%) were considered 3-allele mutants, 2-allele mutants and 1-allele mutants, respectively, although there might be chimera mutants among them (Table 1).

**Table 1:** Table 1. Estimated allele numbers of mutations in the *GWD1* gene in the transformants.

Number of mutant alleles estimated	Transformants	
	Number of mutants	Ratio of mutants (%)
0	26	28
1	35	38
2	20	21
3	10	11
4	2	2
Total	93	100

We analyzed the nucleotide sequences of the 4-allele mutants #88 and #128 and detected four different nucleotide sequences, which corresponded to WT-A to WT-D with genomic nucleotide polymorphisms. Mutant #88 consisted of four mutant sequences, which contained 124-nt deletion and 20-nt insertion, a 439-nt deletion and 55-nt insertion, a 1-nt substitution, and a 26-nt deletion and 1-nt insertion, respectively. Among them, a mutant allele had a 1-nt substitution that occurred at the HindIII site used for CAPS analysis to change the nucleotide sequence AAGCTT to AAACTT. This mutation resulted in changing an alanine residue in the GWD1 protein to a threonine residue. The other three alleles were suggested to be loss-of-function mutations due to frameshifts. Mutant #128 contained four mutant sequences, which contained a 7-nt deletion and a large deletion of more than 400 nt, a 199-nt deletion and 190-nt insertion, a 116-nt deletion, and a 128-nt deletion and 70-nt substitution, all of which were suggested to be loss-of-function mutations (Figure 2B, Supplementary Figure S1B, C).

### Growth of the *GWD1* mutants

**Figure 3:**
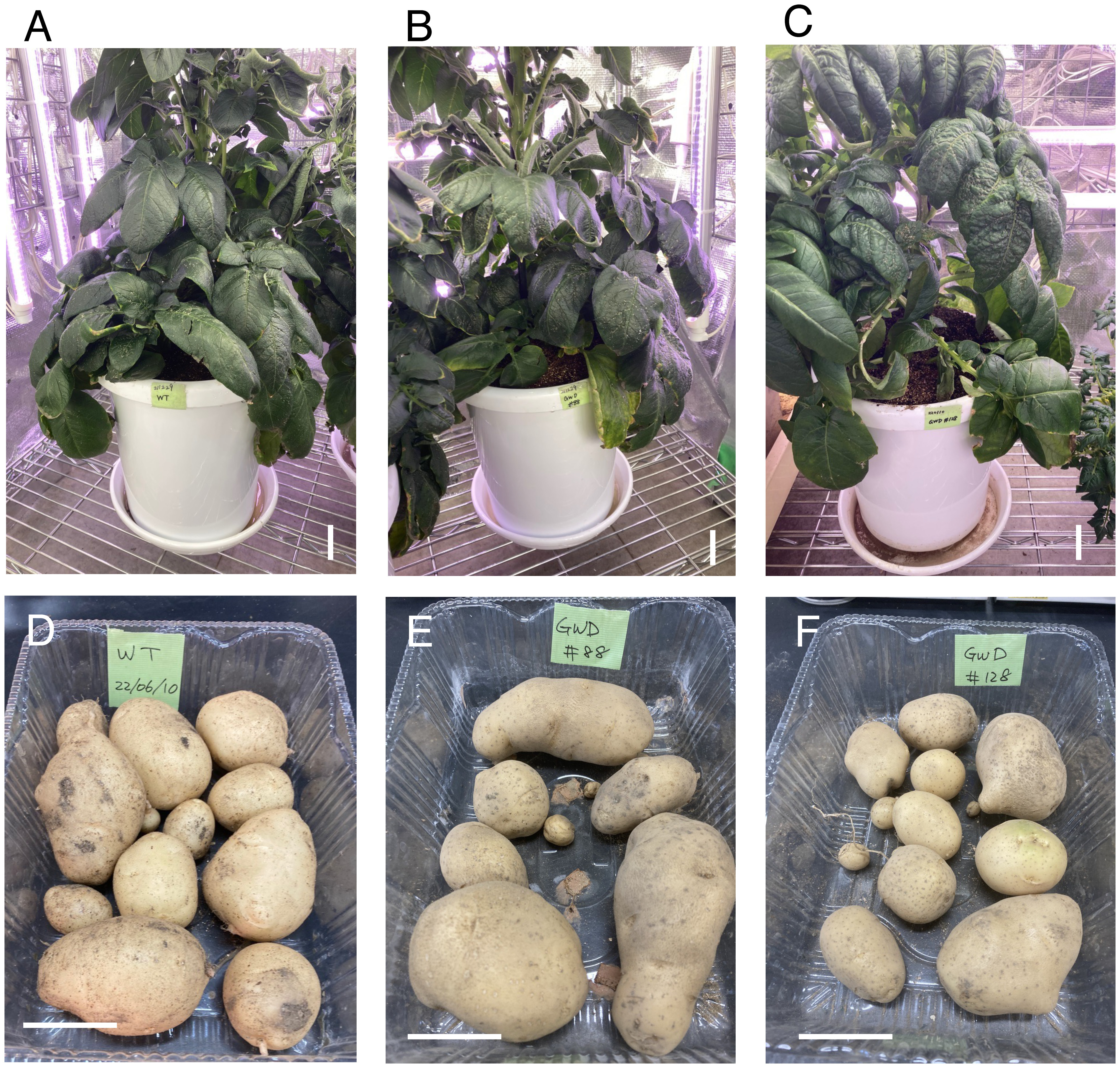
Figure 3. Shapes of the *gwd1* mutant lines. Panels A and D, B and E, and C and F show the plants in the vegetative stage and the harvested tubers of WT, lines #88 and #128, respectively. WT tubers were harvested at 100 days after planting, and those of #88 and #128 were harvested at 120 days after planting. Bars=5 cm.

Genome-edited *gwd1* mutants (#88, #128) were cultivated. Mutant lines #88 and #128 were the 4-allele mutants whose *GWD1* gene was disrupted. They were compared with the wild type (WT, Sayaka) and starch mutant lines, *gbss1* mutant line #F14 and *sbe3* mutant line #19. These *gwd1* mutants normally grew similarly to WT and no difference in plant appearance was found in the vegetative growth phase (Figure 3). However, a delay of growth was observed in #88 and #128 for two or three weeks to reach the reproductive phase. These showed a late flowering feature. These lines flowered in 60–70 days after planting. These mutants showed delayed tuber formation and took longer to grow to harvest. WT, gbss1 #F14 and sbe3 #19 flowered in 50–60 days. Their tubers were harvested at approximately 100 days after planting. On the other hand, tubers formed from the *gwd1* mutants were small, soft and juicy in morphology at that time. After three weeks of more cultivation, these tubers were fully mature and similar to the WT tubers. A sufficient yield of tubers was obtained from the mutants that were harvested at 120 days after planting (Figure 3).

### Properties of the *GWD1* mutant starch

Starch was prepared from the various mutant tubers, including those from the *gwd1* mutant lines #88 and #128, the *gbss1* mutant line #F14- and the *sbe3* mutant line #19. The properties of these starches were determined and compared with those of WT.

**Figure 4:**
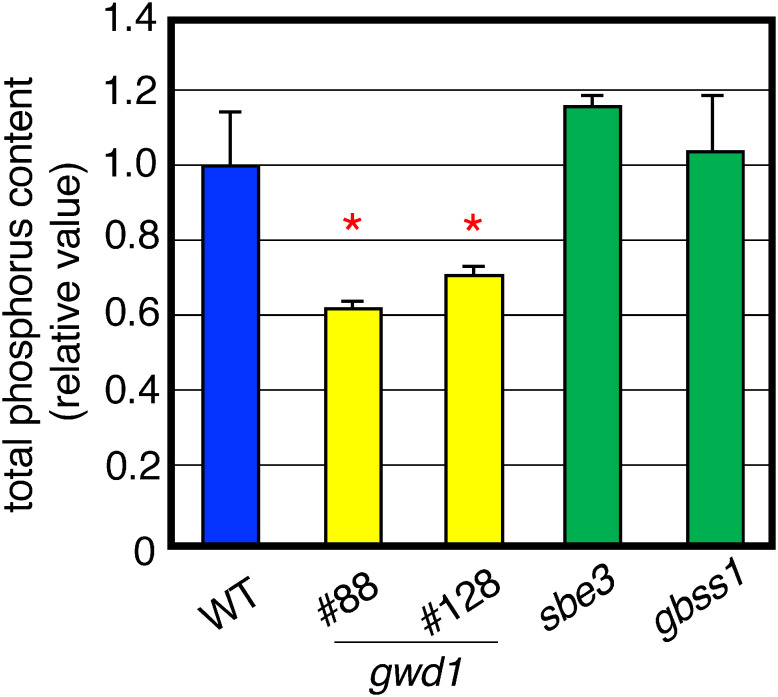
Figure 4. Phosphorus contents in tuber starches prepared from *gwd1* mutants, *sbe3* mutant (#19), *gbss1* mutant (#F14) and wild-type plants. Values are indicated by the relative amounts to that of WT. Values show the average of triplicate measurements. Error bars indicate the standard deviations (*n*=3). Asterisks indicate significant differences in the values of transformants compared to that of wild type at *p*<0.05.

We measured the total phosphorus content in tuber starch. Starch of the *gwd1* mutant lines #88 and #128 was found to have a peculiar trait whose total phosphorus content was significantly less than that of WT (Figure 4). Neither the *gbss1* mutant (#F14) nor the *sbe3* mutant (#19) showed a decrease in phosphorus content (Figure 4). These results suggest that the mutation in the *GWD1* gene caused a decrease in the total phosphorus content in tubers.

The *gwd1* mutants showed significantly higher amylose contents than the WT. The detected amylose contents of mutant lines #88 and #128 were 39.3% and 35.6%, respectively. Those of WT, the *gbss1* mutant (#F14) and the *sbe3* mutant (#19) were 29.2%, 8.6% and 31.9%, respectively (Table 2). These results indicate that these *gwd1* mutants have a significantly high amylose content starch. Gelatinization of these starches was analyzed by DSC. The peak gelatinization temperature (Tp) and the onset of gelatinization temperature (To) were slightly lower than those of WT, whereas and the enthalpy for starch gelatinization (ΔH) was not greatly different between *gwd1* mutant lines #88 and #128 and those of WT (Table 2). This result suggests that the reduction in phosphate content in starch influences the starch gelatinization properties. Starch viscosity was found to greatly differ among the mutant lines. An RVA revealed that both *gwd1* mutant lines, #88 and #128, exhibited obviously lower values of both the peak viscosity and setback than those of WT (Table 2). These observations suggest that the starch of the *gwd1* mutants consists of very low-viscosity starches and is difficult to retrograde. In contrast, the *gbss1* and *sbe3* mutants showed higher peak viscosity with lower setback values and higher peak viscosity with high setback values (Table 2). The RS values were similar to those of WT.

**Table 2:** Table 2. Properties of mutant starches.

Line	Amylose content (%)	DSC			RVA			RS (%)
		Tp (°C)	ΔH (J/g)	To (°C)	Peak viscosity (RVU)	Breakdown (RVU)	Set back (RVU)	
WT	29.2±0.70	73.4±0.12	18.4±0.39	70.6±0.15	105.9±3.93	7.11±1.53	81.9±0.87	72.4±1.20
*gwd1* #88	39.3*±0.96	68.9*±0.06	17.5±0.06	65.4*±0.12	55.2*±0.47	3.92±0.22	15.3*±0.41	75.1±2.41
*gwd1* #128	35.6*±0.95	67.9*±0.27	17.8±0.53	64.6*±0.27	89.3*±0.52	0.95*±0.05	52.7*±0.75	71.7±1.30
*sbe3* #19	31.9±2.00	70.4*±1.02	17.6±0.88	66.3*±1.68	121.2*±5.69	11.20*±2.45	119.2*±3.05	80.7*±0.75
*gbss1* #F14	8.6*±0.85	72.6±0.06	18.8±0.46	68.4*±0.12	153.7*±0.09	72.2*±0.51	5.5*±0.63	76.7±1.31

DSC: thermal properties by differential scanning calorimetry, RVA: pasting properties by rapid visco analyzer, RS: values of resistant starch, Errors indicate ±SD. Asterisks indicate a significant difference compared to WT (*p*<0.01).

### Determination of tissue-released water by freeze–thawing of the mutant tubers

Sections of mutant potato tubers were heated, stored in a freezer, naturally thawed, and wrapped with a high water-absorbing polymer, and the moistures released from tuber sections was measured to determine the ratio of water loss from tuber tissues.

**Figure 5:**
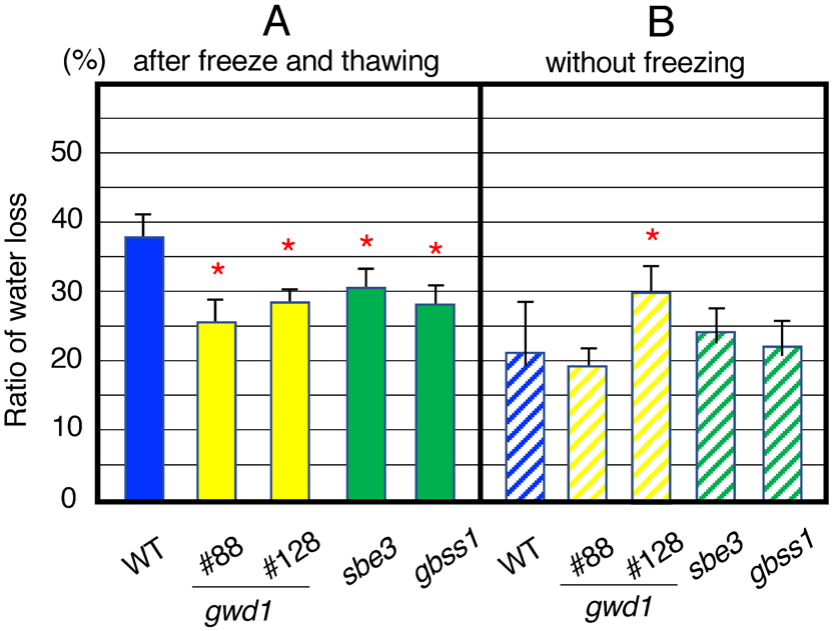
Figure 5. Ratio of tissue-released water from the tubers of *gwd1* mutants, *sbe3* mutant (#19), *gbss1* mutant (#F14) and wild-type plants. (A) Ratios of loss of moisture from tuber sections. Values represent the ratio of lost weights in the tuber sections after freeze–thawing treatment. Error bars indicate the standard deviations (*n*=4). Asterisks indicate significant differences in the values of transformants compared to that of wild type at *p*<0.05. (B) Ratios of loss of moisture from the tuber sections that were tested without freeze–thawing treatment. Values show the ratio of lost weights in the tuber sections due to water absorption by water-absorbing polymer sheets. Error bars indicate the standard deviations (*n*=4). Asterisks indicate significant differences in the values of transformants compared to that of wild type at *p*<0.05.

After the freeze–thaw treatment, 38% of water on average was released from the tuber section of the WT. On the other hand, the ratios of the released water from the tuber sections of *gwd1* mutant lines #88 and #128 were 26% and 28%, respectively. These values were significantly smaller than those of WT (Figure 5A). The *gbss1* mutant (#F14) and the *sbe3* mutant (#19) also showed significantly less amount of released water from the tuber (Figure 5A).

Furthermore, we also determined the amount of water loss from the tuber sections when they were not treated with thawing as the control experiment. After this treatment, the outflow of water was examined. In the results under the without freeze condition, sections of WT and all mutants showed 20–30% water loss, and no significant difference was observed between them other than #128 (Figure 5B). These amounts were nearly comparable to those observed in the mutants lacking GWD1 (#88), GBSS1 and SBE3, suggesting that their tubers have the characteristics of low water loss by freeze–thawing. The *gwd1* #128 line showed similar level of water loss as observed with freeze–thawing treatment, suggesting there might be some difference on its water retention.

## Discussion

Research on starch phosphorylation began with the discovery of the R1 protein, a potato starch-binding protein (Lorberth et al. 1998). A transformant potato repressing R1 gene expression has been reported to show a 90% suppression of the phosphate content in the tuber starch. Its pasting properties reveal a significant decrease in peak viscosity and an increase in setback viscosity (Viksø-Nielsen et al. 2001). The R1 protein is a kinase that accepts glucan chains and catalyzes the phosphorylation of long-chain glucan chains using ATP as a donor and is named α-glucan water dikinase (GWD) (Ritte et al. 2002).

In this study, we created a potato *gwd1* mutant using genome editing technology with CRISPR/dMac3-Cas9. Targeted mutations were carried out in the 7th exon region of the *GWD1* gene and resulted in obtaining two 4-allele mutants (#88, #128). The overall efficiency of genome editing was 72% (Table 1). To date, we have obtained *gbss1* and *sbe3* mutants using the CRISPR/dMac3-Cas9 system (Kusano et al. 2018; Takeuchi et al. 2021). For these mutants, the ratios of mutant generation were 83% and 70%. The efficiency of genome editing of the *GWD1* gene was comparable to that of other mutagenesis experiments.

The proportion of 4-allele mutants was 2% for all mutants, whereas the frequency of the 4-allele mutants was 10% and 8% for generation of the GBSS and the SBE3 mutants, respectively. This efficiency was considered low compared to other cases (Table 1). Because many 2 or 3-allele mutants were obtained in the process of creating *gwd1* mutants, there may be a reason why it was difficult to obtain 4-allele mutants in the genome editing experiment on the *GWD1* gene. This suggests that the *GWD1* gene plays an important role and that defects in it may cause some detrimental effects on potato reproduction.

Both *gwd1* mutant lines #88 and #128 showed similar traits with growth retardation and unique properties on tuber starch that were significantly different from those of WT (Table 2 and Figures 4, 5). The mutant line #128 had deletion/insertion mutations causing frameshift mutations in the *GWD1* gene in all four alleles. Therefore, this mutant was considered a *GWD1* loss-of-function mutant (Figure 2 and Supplementary Figure S1). The mutant line #88 was also a 4-allele mutant, but one mutant allele had a nucleotide substitution, G to A, at the restriction enzyme HindIII site (AAGCTT), which caused an amino acid substitution from GCT (Ala) to ACT (Thr) (Figure 2 and Supplementary Figure S1). This may suggest that the point mutation found in the #88 line significantly contributes to GWD1 function. Alternatively, it is possible that this mutant allele contains an unknown mutation that has resulted in loss of function at this allele gene. However, it is quite unclear why #128 showed weaker phenotype than #88.

Rice *GWD1* has been reported to be involved in rice yield and quality. Overexpression of *GWD1* results in improvement in multiple traits such as yield, grain shape and quality, seed germination, and stress tolerance. This indicates that GWD1 plays an important role in sink tissues (Wang et al. 2021). The importance of starch phosphorylation and breakdown for pollen germination has been suggested in tomato (Nashilevitz et al. 2009). *Arabidopsis* *GWD* mutants are known to exhibit delayed growth and flowering (Yu et al. 2001). Reproductive retardation was also observed in our *GWD1* mutants. This observation suggests that potato GWD1 has a similar function to that of *Arabidopsis*.

The tuber starch of the *gwd1* mutants showed significantly reduced phosphorus content (Figure 4). GWD1 is an enzyme involved in the modification of starch with phosphate groups (Mahlow et al. 2016). Antisense transformants have also shown reduced phosphate content (Wickramasinghe et al. 2009). A large amount of the phosphorus contained in starch is bound to starch as phosphate, and therefore, the *gwd1* mutants are considered to have reduced starch phosphate content.

The *gwd1* mutants showed significantly higher amylose content than the WT. *GWD1* is not a determinant of the amylose and amylopectin biosynthesis. However, in *Arabidopsis thaliana* and *Lotus japonicus*, mutants lacking this gene have been reported to accumulate excessive starch (Caspar et al. 1991; Nashilevitz et al. 2009; Vriet et al. 2010). Viksø-Nielsen et al. (2001) observed an increase in amylose content in *GWD1* knockdown mutants but described that the reason was unknown. The observation of increased amylose content in potato *gwd1* mutants can be ascribed to the overaccumulation of starch as reported in *A. thaliana* and *L. japonicum*.

The starch of the *gwd1* mutants had a very low RVA viscosity (Table 2). These results are consistent with those of previous reports observed on antisense knockdown transformants (Wickramasinghe et al. 2009). Starch of the *gwd1* mutants showed slightly lower gelatinization temperatures than WT starch. Supporting this, the existence of phosphate groups in starch granules is suggested to increase the starch gelatinization temperatures (Kim et al. 1995). Our observations strongly suggest that the *gwd1* mutant starch has a very peculiar and unique features.

Potato tuber starch contains a large amount of phosphorus that is esterified to the amylopectin molecule in starch granules. Phosphate groups in starch bound to moisture function as a water-retaining component. Starch containing many phosphate groups is known to have higher viscosity (Noda et al. 2004a). The phosphate and amylopectin unit chain lengths primarily influence the pasting and rheological properties of starch gels (Blennow et al. 2005). Potato starch with a relatively low phosphorus content is suitable for food processing, such as fish paste products, because syneresis is less likely to occur. On the other hand, an increase in phosphate contained in potato starch is known to improve texture (Noda et al. 2006).

It is known that freezing and thawing processes induce the release of moisture from potato starch gel by syneresis (Freschi et al. 2014). The *gwd1* mutant potato tubers showed less amount of tissue-released water by freeze–thawing treatment (Figure 5). This matter suggests that the decrease in phosphate content in the tuber starch may attribute to reduce syneresis. This property enables the generation of novel potato cultivars that produce starch with low syneresis. The *sbe3* and *gbss1* mutants also showed less amount of tissue-released water by freeze–thawing (Figure 5). In these mutants, structural changes in starch granules occur due to decreased amylose content and changes in amylopectin structure, which may be accompanied by dense structures and increased water retention. Starch with less syneresis contributes to prevention of starch retrogradation and deterioration of quality. These mutants could be useful in food and industrial application.

Our observations showed large differences in the mutant starches due to the deficiency of the targeted genes. Among the genes involved in starch properties, *GWD1* is an ideal target with promising potential for the breeding of elite cultivars with particular and unique starch properties via genome editing.

## Abbreviations

CRISPR/Cas9: clustered regularly interspaced short palindromic repeats-associated protein 9
dMac3-Cas9: Cas9 installed with a translational enhancer dMac3
gRNA: guide RNA
PCR: polymerase chain reaction

## Description of Supplementary Files

Supplementary Figure S1. Nucleotide sequences of the region around the target sites of the *GWD1* gene in the WT-A, WT-B, WT-C and WT-D genomes.

Supplementary Figure S2. CAPS analysis of the regenerated potato lines.
