# Supplementary material for: Peculiar properties of tuber starch in a potato mutant lacking the α-glucan water dikinase 1 gene GWD1 created by targeted mutagenesis using the CRISPR/dMac3-Cas9 system
Source: Plant Biotechnol (Tokyo). 2023 Sep 25;40(3):219–27. doi: 10.5511/plantbiotechnology.23.0823a (PMC10901162; doi:10.5511/plantbiotechnology.23.0823a)
Supplement: Supplementary Data [file plantbiotechnology-40-3-23.0823a-s001.pdf]

A: WT

```
A   acacttctgt tatctactta gttacggata ctgtcagttg tgtatttcag GAGGAATATG
B   acacttctgt tatctactta gttacggata ctgtcagttg tgtatttcag GTGGAATATG
C   acacttctgt tatctactta gttactgata ctgtcagttg tgtatttcag GTGGAATATG
D   acacttctgt tatctactta gttactgata ctgtcagttg tgtatttcag GTGGAATATG

A   AGGCTGCTCG AACTGAGCTA CAGGAGGAAA TAGCTCGTGG TGCTTCCATA CAGGACATTC
B   AGGCTGCTCG AACTGAGCTA CAGGAGGAAA TAGCTCGTGG TGCTTCCATA CAGGACATTC
C   AGGCTGCTCG AACTGAGCTA CAGGAGGAAA TAGCTCGTGG TGCTTCCATA CAGGACATTC
D   AGGCTGCTCG AACTGAGCTA CAGGAGGAAA TAGCTCGTGG TGCTTCCATA CAGGACATTC

A   GAGCAAGGCT AACCAAAACT AATGATAAAA GTCAAAGCAA AGAAGAGCCT CTTCATGTAA
B   GAGCAAGGCT AACCAAAACT AATGATAAAA GTCAAAGCAA AGAAGAGCCT CTTCATGTAA
C   GAGCAAGGCT AACCAAAACT AATGATAAAA GTCAAAGCAA AGAAGAGCCT CTTCATGTAA
D   GAGCAAGGCT AACCAAAACT AATGATAAAA GTCAAAGCAA AGAAGAGCCT CTTCATGTAA

A   CAAAGAGTGA AATACCTGAT GACCTTGCCC AAGCACAAGC TTACATTAGG TGGGAGAAAG
B   CAAAGAGTGA AATACCTGAT GACCTTGCCC AAGCACAAGC TTACATTAGG TGGGAGAAAG
C   CAAAGAGTGA TATACCTGAT GACCTTTCCC AAGCACAAGC TTACATTAGG TGGGAGAAAG
D   CAAAGAGTGA TATACCTGAT GACCTTTCCC AAGCACAAGC TTACATTAGG TGGGAGAAAG

A   CAGGAAAGCC GAACTATCCT CCAGAAAAGC AAATTgtaaa tgctgaactt ttcttacagt
B   CAGGAAAGCC GAACTATCCT CCAGAAAAGC AAATTgtaaa tgctgaactt ttcttacagt
C   CAGGAAAGCC GAACTATCCT CCAGAAAAGC AAATTgtaaa tgctgaacct ttcttacagt
D   CAGGAAAGCC GAACTATCCT CCAGAAAAGC AAATTgtaaa tgctgaacct ttcttacagt

A   ttttgtagtg tgtgtatatg tctaggcgca taaatctggg tattctgtct tctatagatg
B   ttttgtagtg tgtgtatatg tctaggcgca taaatctggg tattctgtct tctatagatg
C   ttttgtagtg tgtgtatatc tccaggcgca taaagctggg tatgctgtct tctatagatg
D   ttttgtagtg tgtgtatatc tccaggcgca taaagctggg tatgctgtct tctatagatg

A   tgagactttg atcaagcatt gttttattaa cagGAAGAAC TCGAAGAAGC AAGAAGAGAA
B   tgagactttg atcaagcatt gttttattaa cagGAAGAAC TCGAAGAAGC AAGAAGAGAA
C   tgagactttg atcaagcatt gttttattaa cagGAAGAAC TCGAAGAAGC AAGAAGAGAA
D   tgagactttg atcaagcatt gttttattaa cagGAAGAAC TCGAAGAAGC AAGAAGAGAA
```

Supplementary Figure S1(A)

B: #88

```
A   acacttctgt tatctactta gttacggata ctgtcagttg tgtatttcag GAGGAATATG
B   ac-------- ---------- ---------- ---------- ---------- ----------
C   acacttctgt tatctactta gttactgata ctgtcagttg tgtatttcag GTGGAATATG
D   acacttctgt tatctactta gttactgata ctgtcagttg tgtatttcag GTGGAATATG

A   AGGCTGCTCG AACTGAGCTA CAGGAGGAAA TAGCT----- ---------- ----------
B   ---------- ---------- ---------- ---------- ---------- ----------
C   AGGCTGCTCG AACTGAGCTA CAGGAGGAAA TAGCTCGTGG TGCTTCCATA CAGGACATTC
D   AGGCTGCTCG AACTGAGCTA CAGGAGGAAA TAGCTCGTGG TGCTTCCATA CAGGACATTC

A   --tttgtaaa tgctgaactt tt(-124 + 20nt insertion)-------- ----------
B   --gacgacta cgcgctaggg ggataagacg agcaaaaagc ttgttaattc gcgctag---
C   GAGCAAGGCT AACCAAAACT AATGATAAAA GTCAAAGCAA AGAAGAGCCT CTTCATGTAA
D   GAGCAAGGCT AACCAAAACT AATGATAAAA GTCAAAGCAA AGAAGAGCCT CTTCATGTAA
      a(insertion)

A   ---------- ---------- ---------- ---------C TTACATTAGG TGGGAGAAAG
B   ---------- ---------- -(-439+55) ---------- ---------- ----------
C   CAAAGAGTGA TATACCTGAT GACCTTTCCC AAGCACAAAC TTACATTAGG TGGGAGAAAG
D   CAAAGAGTGA TATACCTGAT --  (-26+1)  ---------- -------TAGG TGGGAGAAAG

A   CAGGAAAGCC GAACTATCCT CCAGAAAAGC AAATTgtaaa tgctgaactt ttcttacagt
B   ---------- ---------- ---------- ---------- ---------- ----------
C   CAGGAAAGCC GAACTATCCT CCAGAAAAGC AAATTgtaaa tgctgaacct ttcttacagt
D   CAGGAAAGCC GAACTATCCT CCAGAAAAGC AAATTgtaaa tgctgaacct ttcttacagt

A   ttttgtagtg tgtgtatatg tctaggcgca taaatctggg tattctgtct tctatagatg
B   ---------- ---------- ---------- ---------- ---------- ----------
C   ttttgtagtg tgtgtatatc tccaggcgca taaagctggg tatgctgtct tctatagatg
D   ttttgtagtg tgtgtatatc tccaggcgca taaagctggg tatgctgtct tctatagatg

A   tgagactttg atcaagcatt gttttattaa cagGAAGAAC TCGAAGAAGC AAGAAGAGAA
B   ---------- ---------- ---------- ---------- ---------- ----------
C   tgagactttg atcaagcatt gttttattaa cagGAAGAAC TCGAAGAAGC AAGAAGAGAA
D   tgagactttg atcaagcatt gttttattaa cagGAAGAAC TCGAAGAAGC AAGAAGAGAA
```

Supplementary Figure S1.(B)

C: #128

```
A   acacttctgt tatctactta gttacggata ctgtcagttg tgtatttcag GAGGAATATG
B   acacttctgt tatctactta gttacggata ---------- ---------- ----------
C   acacttctgt tatctactta gttactgata ctgtcagttg tgtatttcag GTGGAATATG
D   acacttctgt tatctactta gttactgata ctgtcagttg tgtatttcag GTGGAATATG

A   AGGCTGCTCG AACTGAGCTA CAGGAGGAAA TAGCTCGTGG TGCTTCCATA CAGGA-(-7)
B   ---------- ---------- ---------- ---------- ---------- ----------
C   AGGCTGCTCG AACTGAGCTA CAGGAGGAAA TAGCTCGTGG TGCTTCCATA CAGGACATTC
D   AGGCTGCTCG AACTGAGCTA CAGGAGGAAA TAGCTCGTGG TGCTTCCATA CAGGACATTC

A   --GCAAGGCT AACCAAAACT AATGATAAAA GTCAAAGCAA AGAAGAGCCT CTTCATGTAA
B   ---------- --(-199)-- -(long insertion*+190)---------- ----------
C   G--------- --(-116)-- ---------- ---------- ---------- ----------
D   GA-------- -AGCAGGAAA GCCGAACTAT CCTCCAGAAA AGCAAATTGT AAATGCTGAA

A   CAAAGAGTGA AATACC---- ---------- ---------- ---------- ----------
B   ---------- ---------- ---------- ---------- ------TAGG TGGGAGAAAG
C   ---------- ---------- ---------- ---------- ---------- -------AAG
D   CCTTTCTTAC AGTTTATGTA G--(70 nt) ---------- ---------- ----------

A   ---------- ---------- ---------- ---------- ---------- ----------
B   CAGGAAAGCC GAACTATCCT CCAGAAAAGC AAATTgtaaa tgctgaactt ttcttacagt
C   CAGGAAAGCC GAACTATCCT CCAGAAAAGC AAATTgtaaa tgctgaacct ttcttacagt
D   CAGGAAAGCC GAACTATCCT CCAGAAAAGC AAATTgtaaa tgctgaacct ttcttacagt

A   ---------- ---------- ---------- ---------- ---------- ----------
B   tttagtagtg tgtgtatatg tctaggcgca taaatctggg tattctgtct tctatagatg
C   ttttgtagtg tgtgtatatc tccaggcgca taaagctggg tatgctgtct tctatagatg
D   ---------- tgtgtatatc tccaggcgca taaagctggg tatgctgtct tctatagatg

A   ---------- ---------- ---------- ---------- ---------- ----------
B   tgagactttg atcaagcatt gttttattaa cagGAAGAAC TCGAAGAAGC AAGAAGAGAA
C   tgagactttg atcaagcatt gttttattaa cagGAAGAAC TCGAAGAAGC AAGAAGAGAA
D   tgagactttg atcaagcatt gttttattaa cagGAAGAAC TCGAAGAAGC AAGAAGAGAA
```

```
long insertion* (190 nt)
tttcactctt tgttacatga agaggctctt ctttgctttg acttttatca ttagtttttg
ttagccttgc tcctgtatgg aagcaccacg agctatttcc tcctgtagct cagttcgagc
agcctcatat tcctcctgaa atacacaaca gacagtatcc gtaactaagt agatacccag
atttcttatc
```

Supplementary Figure S1. Nucleotide sequences of the region around the target sites of the *GWD1* gene in the WT-A, WT-B, WT-C and WT-D genomes. Sequences in the exons and introns are shown by upper-case and lower-case letters, respectively. Polymorphic nucleotides between them are highlighted in red. The HindIII site is shown as green letters. Target sites of the gRNAs are underlined and PAM sequences are shown in yellow color. (A) – (C) Nucleotide sequences of WT and mutant lines #88, and #128, respectively. Regions corresponding to the nucleotide deletions are indicated by gaps. A nucleotide substitution in the HindIII site of #88-C is highlighted in yellow color. Insertions are indicated by blue letters.

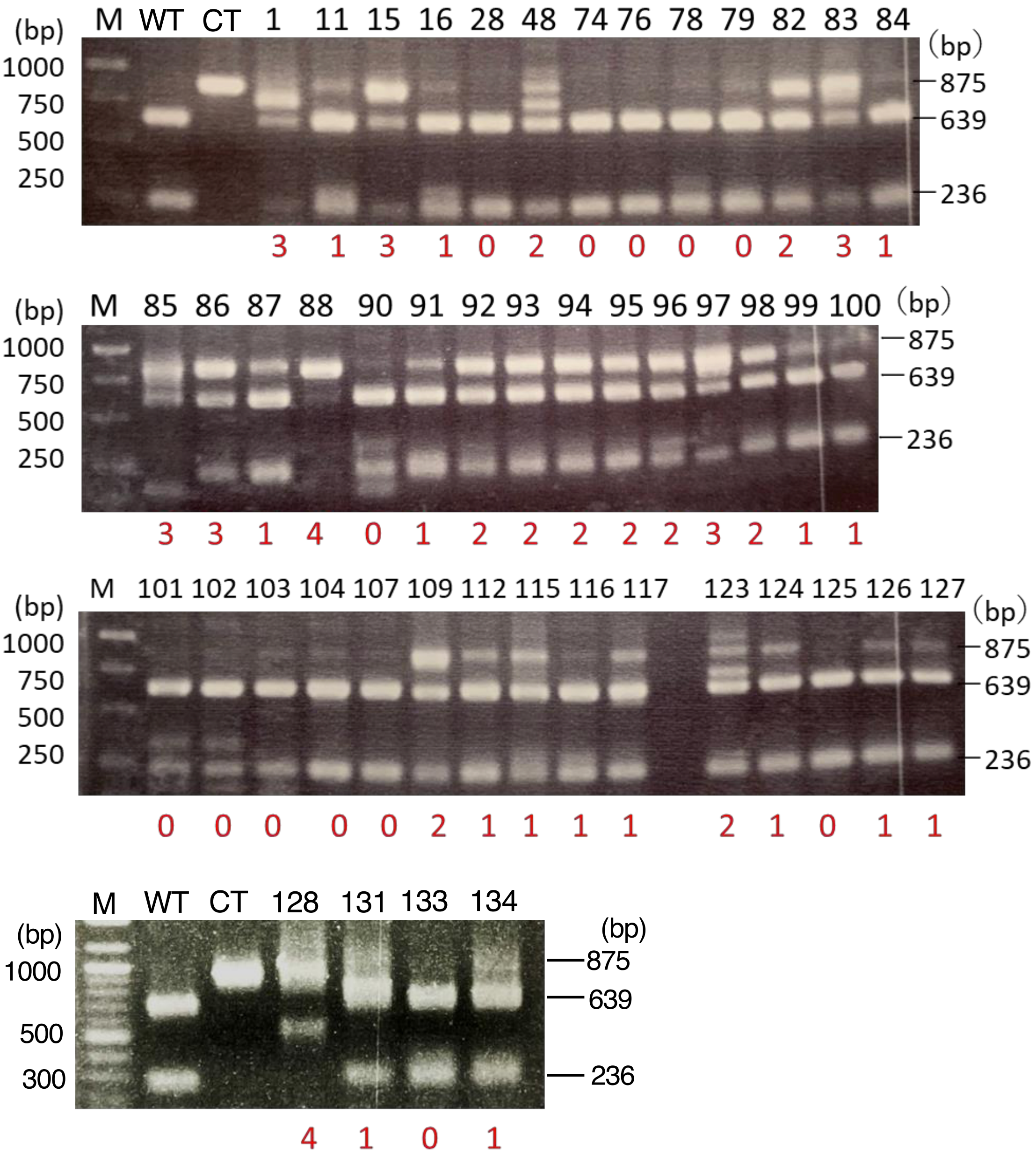

Supplementary Figure S2. CAPS analysis of the regenerated potato lines. HindIII-digested PCR-amplified fragments are indicated. Representative lines are shown. Numbers with the prefix # indicate the names of transformant lines. M: size marker, CT: PCR-amplified fragment of the region around the target site in the wild-type gene, and WT: PCR-amplified and HindIII-digested WT fragment. Predicted numbers of mutant alleles of the *GWD1* gene are indicated below the figure.
